# Small area variations and factors associated with blood pressure and body-mass index in adult women in Accra, Ghana: Bayesian spatial analysis of a representative population survey and census data

**DOI:** 10.1371/journal.pmed.1003850

**Published:** 2021-11-11

**Authors:** Sierra N. Clark, James E. Bennett, Raphael E. Arku, Allan G. Hill, Günther Fink, Richard M. Adanu, Richard B. Biritwum, Rudolph Darko, Ayaga Bawah, Rosemary B. Duda, Majid Ezzati

**Affiliations:** 1 Department of Epidemiology and Biostatistics, Imperial College London, London, United Kingdom; 2 MRC Centre for Environment and Health, Imperial College London, United Kingdom; 3 Department of Environmental Health Sciences, School of Public Health and Health Sciences, University of Massachusetts, Amherst, Massachusetts, United States of America; 4 Department of Social Statistics and Demography, University of Southampton, Southampton, United Kingdom; 5 Department of Epidemiology and Public Health, Swiss Tropical and Public Health Institute and University of Basel, Basel, Switzerland; 6 Department of Population, Family, and Reproductive Health, University of Ghana, Accra, Ghana; 7 Department of Community Health, University of Ghana, Accra, Ghana; 8 School of Medicine, University of Ghana, Accra, Ghana; 9 Regional Institute for Population Studies, University of Ghana, Accra, Ghana; 10 Harvard Medical School, Harvard University, Boston, Massachusetts, United States of America; 11 Abdul Latif Jameel Institute for Disease and Emergency Analytics, Imperial College London, London, United Kingdom; Harvard University, UNITED STATES

## Abstract

**Background:**

Body-mass index (BMI) and blood pressure (BP) levels are rising in sub-Saharan African cities, particularly among women. However, there is very limited information on how much they vary within cities, which could inform targeted and equitable health policies. Our study aimed to analyse spatial variations in BMI and BP for adult women at the small area level in the city of Accra, Ghana.

**Methods and findings:**

We combined a representative survey of adult women’s health in Accra, Ghana (2008 to 2009) with a 10% random sample of the national census (2010). We applied a hierarchical model with a spatial term to estimate the associations of BMI and systolic blood pressure (SBP) and diastolic blood pressure (DBP) with demographic, socioeconomic, behavioural, and environmental factors. We then used the model to estimate BMI and BP for all women in the census in Accra and calculated mean BMI, SBP, and DBP for each enumeration area (EA). BMI and/or BP were positively associated with age, ethnicity (Ga), being currently married, and religion (Muslim) as their 95% credible intervals (95% CrIs) did not include zero, while BP was also negatively associated with literacy and physical activity. BMI and BP had opposite associations with socioeconomic status (SES) and alcohol consumption. In 2010, 26% of women aged 18 and older had obesity (BMI ≥ 30 kg/m^2^), and 21% had uncontrolled hypertension (SBP ≥ 140 and/or DBP ≥ 90 mm Hg). The differences in mean BMI and BP between EAs at the 10th and 90th percentiles were 2.7 kg/m^2^ (BMI) and in BP 7.9 mm Hg (SBP) and 4.8 mm Hg (DBP). BMI was generally higher in the more affluent eastern parts of Accra, and BP was higher in the western part of the city. A limitation of our study was that the 2010 census dataset used for predicting small area variations is potentially outdated; the results should be updated when the next census data are available, to the contemporary population, and changes over time should be evaluated.

**Conclusions:**

We observed that variation of BMI and BP across neighbourhoods within Accra was almost as large as variation across countries among women globally. Localised measures are needed to address this unequal public health challenge in Accra.

## Abstract 2

Sierra N. Clark and colleagues analyze spatial variations of blood pressure and body mass index, and associated factors in Ghanaian women.

## Author summary

**Why was this study done?:**

- The social, physical, and food environment influence the spatial distribution of noncommunicable diseases and their risk factors such as blood pressure (BP) and body-mass index (BMI) within and across neighbourhoods in cities.
- In sub-Saharan Africa (SSA), where cities are growing rapidly and BMI and BP have risen, there is limited information on within-city variation in cardiometabolic risk factors.
- This information could inform targeted and equitable public health programmes.

**What did the researchers do and find?:**

- We combined data from an epidemiological study that had randomly sampled women in Accra and geospatial data on roads and community biomass fuel use to understand how individual and household factors as well as residence predict BMI and BP.
- Data were analysed in a Bayesian spatial model and then used together with the data from the census to predict BP and BMI levels for all women in Accra.
- Local area variation in BMI and BP is large within Accra, similar to patterns seen in other cities and regions around the world.
- Average BMI was higher among women with higher socioeconomic status (SES), as measured by ownership of assets and household infrastructure.

**What do these findings mean?:**

- The spatial variations in BMI and BP across neighbourhoods in Accra were almost as high as variation seen between countries.
- Localised public health actions can help address these disparities and reduce the resulting overall public health burden.

## Introduction

Elevated blood pressure (BP) and body-mass index (BMI) are important risk factors for noncommunicable diseases (NCDs), such as cardiovascular diseases (CVDs), diabetes, kidney disease, and dementia [1,2], and responsible for substantial burden of NCDs [3]. While high-income countries have experienced declines in population levels of BP and hypertension over the years, BP and hypertension in sub-Saharan Africa (SSA), the fastest urbanising region of the world [4], is currently rising and at levels surpassing high-income countries [5,6]. BMI is similarly rising in SSA, particularly among women [7,8]. Within SSA, raised BP and BMI appear to be more prevalent in cities compared to rural areas [9–11]. Urban women in SSA are the only group in the world whose average BMI is rising faster than their rural counterparts [11].

Cities can facilitate health promotion and disease prevention [12]. However, inequalities across neighbourhoods and social groups in access to diverse foods, quality healthcare, recreational and sports spaces, and clean and healthy environments are substantial [12,13] and may lead to variable distribution of NCDs in urban areas [14]. Information on which individuals and communities are at risk in cities can help target public health NCD interventions to where the need is largest.

Previous research has highlighted urban–rural differences in BMI and BP in SSA and individual socioeconomic determinants and inequalities [9–11,15–17]. In addition, a previous study spatially modelled overweight and obesity for women across subregions in Ghana [18]; however, there is very limited information on within-city spatial variations in BMI and BP at the small area level in SSA. We used data from a representative health survey of adult women’s health in Accra, Ghana and a national census to understand factors associated with BMI and BP and map the predicted levels across census enumeration areas (EAs) within the Accra Metropolitan Area (AMA).

## Methods

### Ethics

The 10% random sample of the national Ghana census is freely and publicly available online (https://statsghana.gov.gh). The original WHSA study protocol and data collection was approved by the Harvard University Institutional Review Board in 2003. The current analysis protocol was approved by the Imperial College London ethics committee in 2018 (18IC4885).

### Study area and population

More than 50% of the population in Ghana now lives in urban areas [19]. AMA, the capital and largest city (estimated population of 1.66 million in the 2010 national census) [20], has become a hub in Africa for business, technology, and education. In 2010, half of the residents were younger than 24 years old, and 89% of individuals aged 12 years and older were literate [20]. Previous studies have highlighted that Accra is at the crossroad of epidemiological transition, as the population is experiencing a burden of chronic and infectious diseases [21]. However, social and spatial disparities in access to basic urban resources and services exist in Accra [19], which can create health inequalities. The highest prevalence of obesity in Ghana is in the Greater Accra Region (25%), although little is known about within-city variations and inequalities of obesity and other risk factors for NCDs [22].

### Data sources

We used data from three sources: the Women’s Health Study of Accra (WHSA) survey, the 2010 national census, and a shapefile of the Accra road network [23].

**Fig 1:**
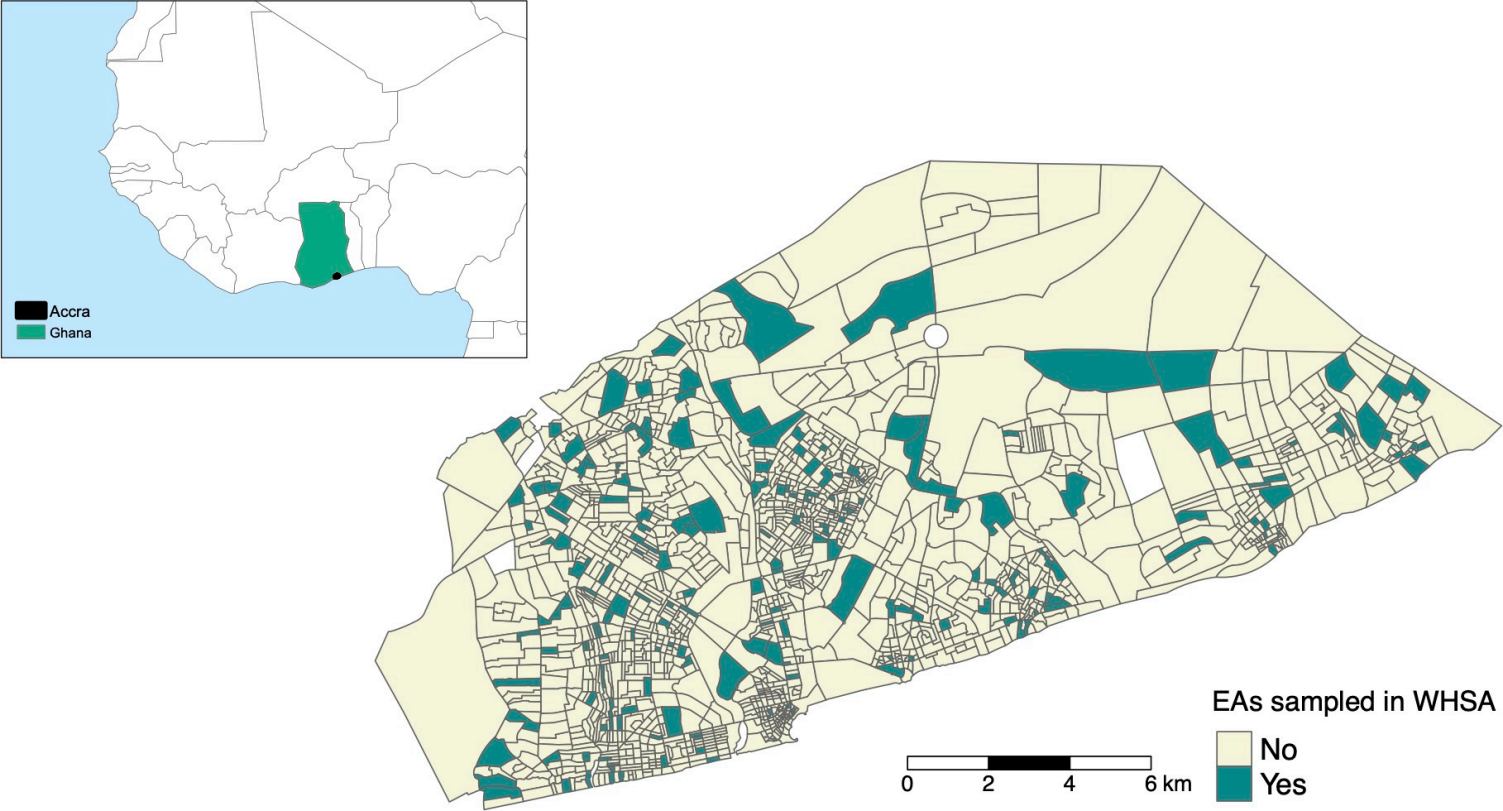
Census (year 2000) EAs where women were sampled for the WHSA (2008 to 2009) in the AMA, Ghana. Accra and EA boundaries are from the Ghana Statistical Service, and the background map of Africa and Ghana in the inset are made with Natural Earth vector map data: https://www.naturalearthdata.com/downloads/110m-cultural-vectors/. AMA, Accra Metropolitan Area; EA, enumeration area; WHSA, Women’s Health Study of Accra.

We used community-based cross-sectional survey data, which are representative of adult women living in Accra. The data were collected in 2008 to 2009 as part of the WHSA study [24–27]. The sampling scheme of the WHSA survey (WHSA wave 2) built on the first WHSA survey which was conducted in 2003 (WHSA wave 1). For the first survey, 3,200 women (≥18 years old) from 200 EAs were originally sampled (Fig 1) to be representative of the adult female population in Accra with a 2-stage cluster probability sampling scheme based on socioeconomic status (SES) and age, following full listing of all households in the 200 EAs selected [27]. The number of women enrolled was calculated as described in [24], and the study achieved a high response rate (3,183 of 3,200 women surveyed). Women older than 55 years were also oversampled to ensure sufficient sample size for age-specific analysis [25]. The second survey conducted in 2008 to 2009, which is used in this present analysis, consisted of 2,814 women. Women who could not be followed up from wave 1 were replaced with new participants (995 women) of equivalent age and SES [27].

BP was measured in participants’ homes with a battery-operated automated sphygmomanometer. Two cuff sizes were available, and women sat in a resting upright position. Four BP readings were taken on the same arm (left or right), and the average of the last three was used for analysis. With lightweight clothing and no shoes, height was measured to the nearest 0.5 cm and weight on a calibrated scale to the nearest 0.1 kg. The WHSA gathered information on demographic and socioeconomic variables, the living environment, and health behaviours, which included, but went beyond, those captured in the census. Prior to model building, we omitted women in the WHSA from the analysis who self-reported as pregnant at the time of survey (*n* = 79). We also restricted the census dataset to women who were over 18 years of age to match the WHSA survey participants.

We obtained and used a 10% random sample of the 2010 National Population and Housing Census. In deviation from our original protocol, we restricted the analyses to the more recent 2010 census and did not use the 2000 census, which has used different EAs. The sample was taken from the full census data such that 10% of households were randomly sampled within each census EA. EAs are small geographical units with a median population of 800 people, 225 households, and area of 0.03 km^2^ within AMA (based on the 2010 census boundaries). The census includes individual- and household-level variables related to demographics, socioeconomic factors, and living environment.

We obtained shapefiles of EAs for the 2010 and 2000 censuses for Accra from the Ghana Statistical Service. Since the 2010 geographic boundary of the Greater AMA was larger than the 2000 census boundary, which was used for sampling in the WSHA study (Fig 1), we used a subset of the area of 2010 that coincided with the geographic extent of the 2000 census where WHSA participants lived. The resulting study area (275 km^2^) included 2,634 EAs. Finally, we used a road network shapefile [23] and extracted all major roads (e.g., motorways) as a proxy for road traffic air and noise pollution.

### Statistical analysis

The analytical aims of our study were to estimate the associations of individual, household, and area-level factors with BMI, systolic blood pressure (SBP) and diastolic blood pressure (DBP) among women who participated in the WHSA accounting for spatial autocorrelation, to predict BMI and BP for all women in the 10% sample of the census data, and to use the predictions to map mean BMI and BP for each EA.

All independent and dependent variables were inspected using univariate summaries and graphs to check for implausible values. Additionally, we assessed dependent variables for normality in their distribution. For some independent variables, we combined variable categories in order to harmonise between the census and the WHSA; for other variables, we used categories that are relevant for health (e.g., cooking fuel as whether biomass fuels or not [28,29]). Missing data were generally uncommon, and observations were dropped from the analysis if missing one or more variables. Nine women from the WHSA were omitted because they did not have a BP and a BMI measurement. There were less than 2% of women missing data from relevant variables in the WHSA (*n* = 58) and 3.8% in the 2010 census (*n* = 2,901).

Summary statistics, including arithmetic means, proportions, and measures of variance, were first generated for all variables evaluated in the statistical models (Table 1) for the WHSA and census separately. The demographics were generally similar between the census and WHSA participants in Accra, but the WHSA participants had a higher percentage of women who were of Ga ethnicity, illiterate, and older than 55 years compared with the 10% random sample of the 2010 census (Table 1). Older women were by design oversampled in the WHSA wave 1 and wave 2, which explains the difference in age structure and likely also explains differences in literacy. The overrepresentation of Ga women in the WHSA was not intentional in the original study sampling design and may have been because EAs along the coast of Accra where the Ga traditionally live were in the sample.

**Table 1:** Summary statistics of SBP (mm Hg), DBP (mm Hg), BMI (kg/m^2^), and demographic, behavioural, socioeconomic, and environmental factors in the WHSA and for women of similar ages living in Accra in the 10% random sample of the 2010 Ghana census.

	WHSA (2008 to 2009)	10% census (2010)
	Adult nonpregnant women with BP and/or BMI measurements* in Accra	Adult women in Accra
**Total number of women**	2,668	72,371
**SBP** (unweighted mean (SD))	130.7 (23.9)	-
**DBP** (unweighted mean (SD))	83.9 (14.7)	-
**BMI** (unweighted mean (SD))	28.4 (6.9)	-
**Age** (*n* (%))		
18 to 24	733 (27)	17,532 (24)
25 to 34	567 (22)	22,517 (31)
35 to 54	697 (26)	22,873 (32)
55+	671 (25)	9,449 (13)
**Literate** (*n* (%))		
Yes	1,813 (68)	61,277 (85)
**Currently married** (*n* (%))		
Yes	1,282 (48)	30,968 (43)
**Religion** (*n* (%))		
Christian	2,141 (81)	60,061 (83)
Muslim	332 (12)	9,754 (13)
Other or no religion	195 (7)	2,556 (4)
**Ethnicity** (*n* (%))		
Ga	1,057 (40)	19,668 (27)
Fante	335 (13)	8,426 (12)
Ewe	378 (14)	12,372 (17)
Other	898 (33)	31,905 (44)
**Household uses biomass as primary cooking fuel** (*n* (%)) Yes	1,640 (61)	37,684 (52)
**Smoke tobacco** (*n* (%)) Yes	22 (<1)	-
**Has ever drank alcohol** (*n* (%)) Yes	1,410 (53)	-
**Spends majority of workday sedentary** (*n* (%)) Yes	2,462 (92)	-
**SES PC1** (*n* (%))**		
First quantile (low)	512 (19)	14,475 (20)
Second quantile	577 (22)	14,474 (20)
Third quantile	521 (19)	14,474 (20)
Fourth quantile	468 (18)	14,474 (20)
Fifth quantile (high)	590 (22)	14,474 (20)
**SES PC2** (*n* (%))**		
First quantile (low)	277 (10)	14,475 (20)
Second quantile	494 (19)	14,474 (20)
Third quantile	654 (24)	14,474 (20)
Fourth quantile	638 (24)	14,474 (20)
Fifth quantile (high)	605 (23)	14,474 (20)
**Distance of EA of residence (centroid) to nearest major road in kilometres** (median (IQR))	0.67 (0.33, 1.30)	0.66 (0.31, 1.35)

Estimates and spatial distribution of diabetes prevalence among WHSA women are presented in Fig F in S1 Text.

* Final sample of WHSA women included in the models: Women were omitted from the analysis if they were pregnant at the time of the survey, did not have SBP, DBP, and BMI measurements and if they were missing independent variable information to be used in the models.

** Quantile breaks based off the distribution of PC1 and PC2 from the 2010 census. PC1 is the primary SES indicator.

%, percentage; BP, blood pressure; BMI, body-mass index; DBP, diastolic blood pressure; EA, enumeration area; IQR, interquartile range; mean, arithmetic mean; *n*, number of observations; PC, principal component; SBP, systolic blood pressure; SD, standard deviation; SES, socioeconomic status; WHSA, Women’s Health Study of Accra.

The models were Bayesian and spatial in structure. Models were run at the individual level and included random intercepts for EAs to account for unmeasured area variations on BMI and BP. Specifically, the observed measurement of SBP, DBP, or BMI for each woman *i*, living in EA *j*, is given by *Y_i_* and modelled as follows:

$$Y_{i}=\beta_{0}+\phi_{j}+\sum_{c=1}^{c=n}\beta_{c}∙{Covariate}_{ci}+\varepsilon_{i},$$

where *β*_0_ is the overall intercept, *ϕ_j_* is the spatial term for EA *j*, *β_c_* is the coefficient for covariate *c*, *Covariate_ci_* is the value of covariate *c* for woman *i*, and *n* is the total number of independent variables in the model. The term *ε_i_* accounts for the variance not captured by other terms and is specified with a Gaussian distribution with variance *σ*^2^.

We used a conditional autoregressive (CAR) prior on the random intercepts in order to allow neighbouring EA’s to share information as unmeasured effects are likely to vary smoothly in space [30]. The spatial random effect for EA *j*, *ϕ_j_*, is specified via an intrinsic CAR prior

$$\phi_{j}|\phi_{-j}∼N(\phi_{j}^{*},\frac{\sigma_{\phi }^{2}}{m_{j}}),$$

where $\phi_{j}^{*}$ is the mean of the ϕ_*j*_ of the contiguous neighbours of EA *j*, and *m_j_* is the number of contiguous neighbours of EA *j*. Neighbouring EAs, irrespective of size, were assigned equal weights.

We used weakly informative priors so that inference on the parameters was driven by the data. Priors for the residual and spatial random effects variances were specified via wide uniform distributions on the standard deviation scale, *σ*^2^~U[0,20] and $\sigma_{\phi }^{2}∼U[0,10]$, respectively. We used N(0,10000) as the prior for the overall intercept and coefficients for the covariates. We used Markov chain Monte Carlo (MCMC) methods to obtain samples from the posterior distribution of the parameters in the models. We ran the models with 2 chains until convergence to a stationary distribution. Convergence of the chains was visually assessed with trace plots. Diagnostic checks on residuals were conducted including checking for normality in distribution, random, and constant variance across fitted values, spatial distribution of residuals across EAs, and influential observations. We assessed the predictive accuracy of the models using mean error (ME), which measures the extent of bias in predictions, and median absolute error (MAE), which measures any deviation in predictions. We report these metrics for the entire samples as well as stratified by age group.

We first estimated the associations of individual-level BMI and BP with demographic, socioeconomic, behavioural, and environmental variables that were determined a priori from previous studies [17,31–34]. We present the associations for a core and extended set of independent variables in the models. The core set of variables are those that were measured both in the WHSA and the census and included: age (years), marital status (currently married yes/no), literacy (literate yes/no), ethnicity (Ga, Fante, Ewe, and other), religion (Christian, Muslim, and other), relative SES index (principal component analysis based on household assets; see Fig A in S1 Text), and proxies of environmental exposures including household primary use of biomass for cooking (yes/no; proxy for women’s exposure to household air pollution), percentage of households in each EA cooking primarily with biomass fuels (proxy for woman’s exposure to community air pollution from biomass burning), and distance of EA centroid to nearest major road (km; proxy for exposure to road-traffic air and noise pollution). The extended set comprised of the core variables plus additional factors that were collected in the WHSA but not the census, including alcohol consumption (ever) (yes/no), current tobacco smoker (yes/no), sedentary for most of the workday (yes/no), and moderate and/or vigorous physical activity for leisure during a typical week (yes/no). In addition, the BP models with the extended set of variables included BMI as an independent variable. For the second step in the analysis, we used the models with the core set of variables to predict BMI and BP levels for women in the 2010 census in Accra.

We present the mean posterior associations of the core and extended set of independent variables with BMI and BP with 95% credible intervals (95% CrIs) around the mean estimate. We also report the posterior probability (PP) of excluding zero for that association. Model predictions of BMI, SBP, and DBP were summarised from the posterior samples as means with 95% CrIs for all women in the city and stratified by age groups. We further calculated the percentage of women who had obesity (BMI ≥ 30 kg/m^2^) as per the World Health Organization’s (WHO) criteria and the percentage of women with uncontrolled hypertension (SBP ≥140 mm Hg and/or DBP ≥90 mm Hg) in Accra [35]. Uncontrolled hypertension is also referred to as having raised SBP and/or DBP, following WHO Global Monitoring Framework for Non-Communicable Diseases [5,35]. Uncontrolled hypertension includes those women taking medication for hypertension (20% of the WHSA sample) but with BP levels above the target [5,36]. We note, however, that in a clinical setting, diagnosis of hypertension tends to involve measurements conducted over multiple visits which is different from health surveys when measurements are taken at a single occasion. For each EA, we calculated the mean levels of BMI and BP and the percentage of women with uncontrolled hypertension and obesity. Finally, we also calculated the probability that mean BMI and BP levels were greater than the city-wide mean based on posterior samples, which takes into account uncertainty in the predictions.

### Software

Data preparation, cleaning, and visualisation was done in *R* (version 3.5.1). One map was created using ArcGIS software by Esri. All models were implemented in *WinBUGS* (version 1.4.3).

### Reporting

This study is reported as per the Strengthening the Reporting of Observational Studies in Epidemiology (STROBE) guideline (S1 STROBE Checklist).

## Results

### Associations of BMI and BP with demographic, socioeconomic, behavioural, and environmental variables

**Fig 2:**
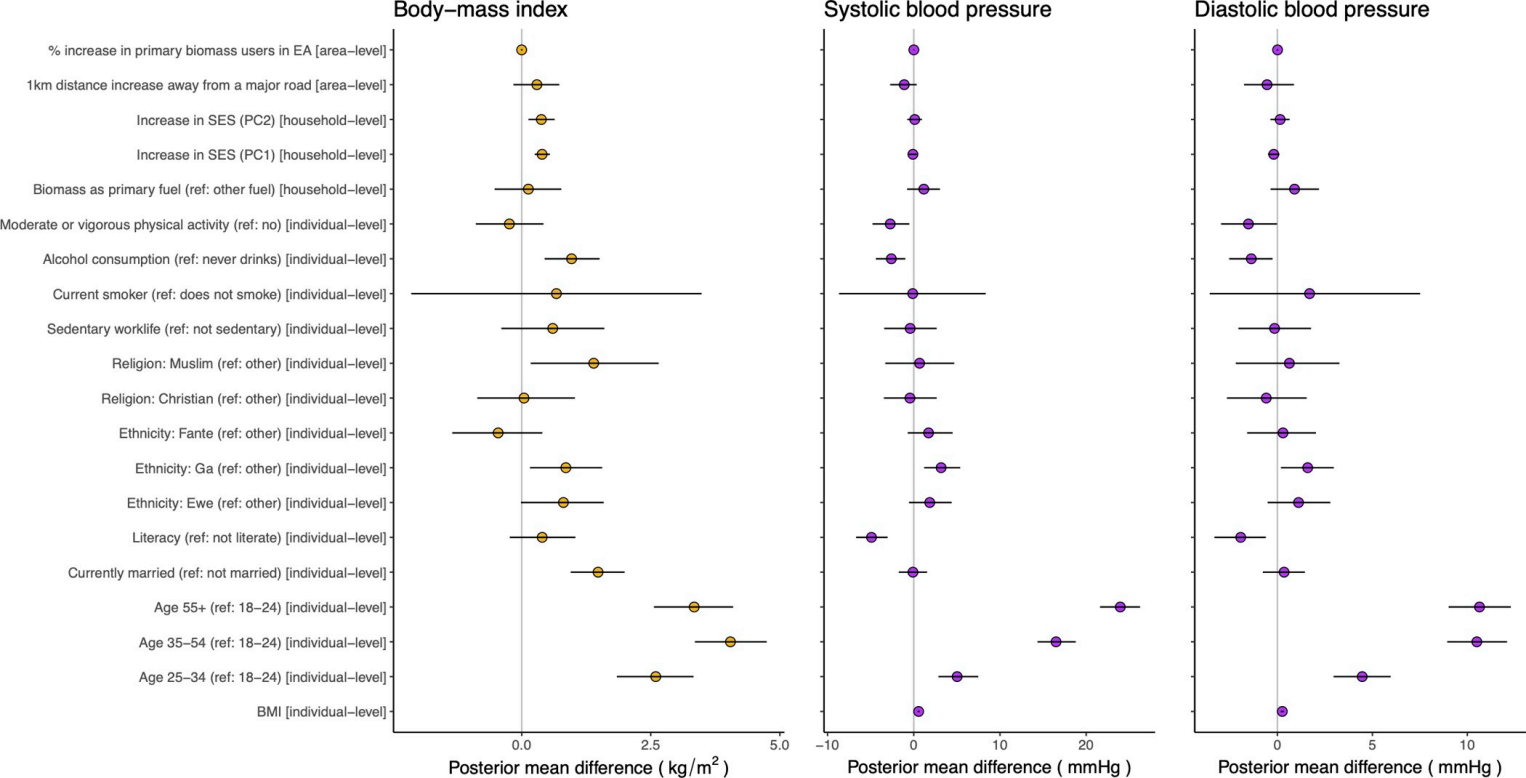
Associations of BP and BMI with demographic, behavioural, socioeconomic, and environmental factors in the WHSA. The figure includes the extended variable set. For each variable, the figure shows the posterior mean association and the 95% CrI. Associations among the core variable set are in Fig B in S1 Text. The prevalence of women who smoke tobacco in Accra was very low (<1% in WHSA), resulting in the large uncertainty around the mean associations. 95% CrI, 95% credible interval; BMI, body-mass index; BP, blood pressure; EA, enumeration area; PC 1 and 2, principal components 1 and 2; ref, reference for categorical variables; SES, socioeconomic status; WHSA, Women’s Health Study of Accra.

Among the extended set of variables, BP was positively associated with age (PP ≥ 0.99), ethnicity (Ga, ref other) (PP ≥ 0.99), BMI (PP ≥ 0.99) and negatively associated with literacy (PP ≥ 0.99), alcohol consumption (PP ≥ 0.99), and moderate and/or vigorous physical activity (PP ≥ 0.99), as the 95% CrIs did not include zero (Fig 2). BMI was positively associated with age (PP ≥ 0.99), alcohol consumption (PP ≥ 0.99), ethnicity (Ga, ref other) (PP = 0.98), relative SES (PP ≥ 0.99), religion (Muslim, ref other) (PP = 0.99), and marital status (PP ≥ 0.99). The associations of BMI and BP with physical activity were both in the negative direction, although the 95% CrI for BMI included zero (PP = 0.73). Several variables had opposite associations in the BMI versus BP models. For example, literacy was weakly positively associated with BMI (PP = 0.90) but negatively associated with BP; SES (first and second principal components) was positively associated with BMI and weakly negatively associated with DBP (first principal component) (PP = 0.88); alcohol consumption was positively associated with BMI and negatively associated with BP. BMI and BP were not significantly associated with proxy variables for indoor and outdoor air and noise pollution as their 95% CrIs included zero. Although in some cases, such as SBP and distance away from major road (PP = 0.93) and DBP and biomass fuel use (PP = 0.90), the 90% credible intervals did not include zero. Associations of BMI and BP with the core set of variables used for prediction were similar to the extended variable set (Fig B in S1 Text).

### Spatial patterns of BMI and BP among women in Accra in 2010

City-wide mean predicted BMI, SBP, and DBP for adult women were 28 kg/m^2^ (95% CrI 28, 29), 128 mm Hg (95% CrI 127, 129), and 83 mm Hg (95% CrI 82, 84), respectively. An estimated 26% (95% CrI 21, 31) of women were obese, and 21% (95% CrI 17, 24) had uncontrolled hypertension (Table 2). Moreover, 8% of women were classified as both obese and having uncontrolled hypertension. Among women aged 18 to 24 years old in Accra, less than 1% were obese and/or had uncontrolled hypertension. Overall mean BMI and BP among WHSA women were slightly higher than the mean of the modelled predictions among census women, possibly because older women had been oversampled in the WHSA (Table A in S1 Text).

**Table 2:** Predicted SBP, DBP, BMI, and percentage of women classified as having obesity and uncontrolled hypertension among adult Accra women in the 2010 census (10% random sample) from hierarchical spatial Bayesian models.

	Mean (95% CrI)			Percent above cutoff (95% CrI)	
Age range	BMI kg/m^2^	SBP mm Hg	DBP mm Hg	Obesity %	Uncontrolled hypertension %
**All ages**	28.1 (27.8, 28.5)	127.6 (126.5, 128.6)	83.0 (82.3, 83.8)	26% (21, 31)	21% (17, 24)
18 to 24	25.1 (24.5, 25.6)	115.9 (114.3, 117.6)	75.8 (74.5, 76.8)	<1% (0, 2)	<1% (0, 1)
25 to 34	28.2 (27.6, 28.7)	122.4 (120.7, 124.3)	81.2 (79.9, 82.4)	18% (9, 26)	<1% (0, 2)
35 to 54	30.0 (29.5, 30.5)	135.1 (133.5, 136.8)	87.7 (86.7, 88.7)	51% (41, 61)	31% (22, 42)
55+	29.0 (28.4, 29.5)	143.7 (141.9, 145.2)	88.2 (86.9, 89.6)	30% (20, 40)	80% (70, 90)

Summaries from the WHSA survey are presented in Table A in S1 Text.

Obesity: BMI ≥ 30 kg/m^2^, uncontrolled hypertension: SBP ≥ 140 mm Hg, and/or DBP ≥ 90 mm Hg.

95% CrI, 95% credible interval; BMI, body-mass index; DBP, diastolic blood pressure; SBP, systolic blood pressure; WHSA, Women’s Health Study of Accra.

There were inequalities in mean BMI and BP across EAs within Accra. The differences in mean levels between EAs at the 10th and 90th percentiles of the distribution was 2.7 kg/m^2^ (BMI), 7.9 mm Hg (SBP), and 4.8 mm Hg (DBP). Similarly, the EAs at the 10th and 90th percentiles of the distribution of the prevalence of women with obesity and uncontrolled hypertension had prevalence’s ranging from 4% to 50% and 3% to 32%, respectively.

**Fig 3:**
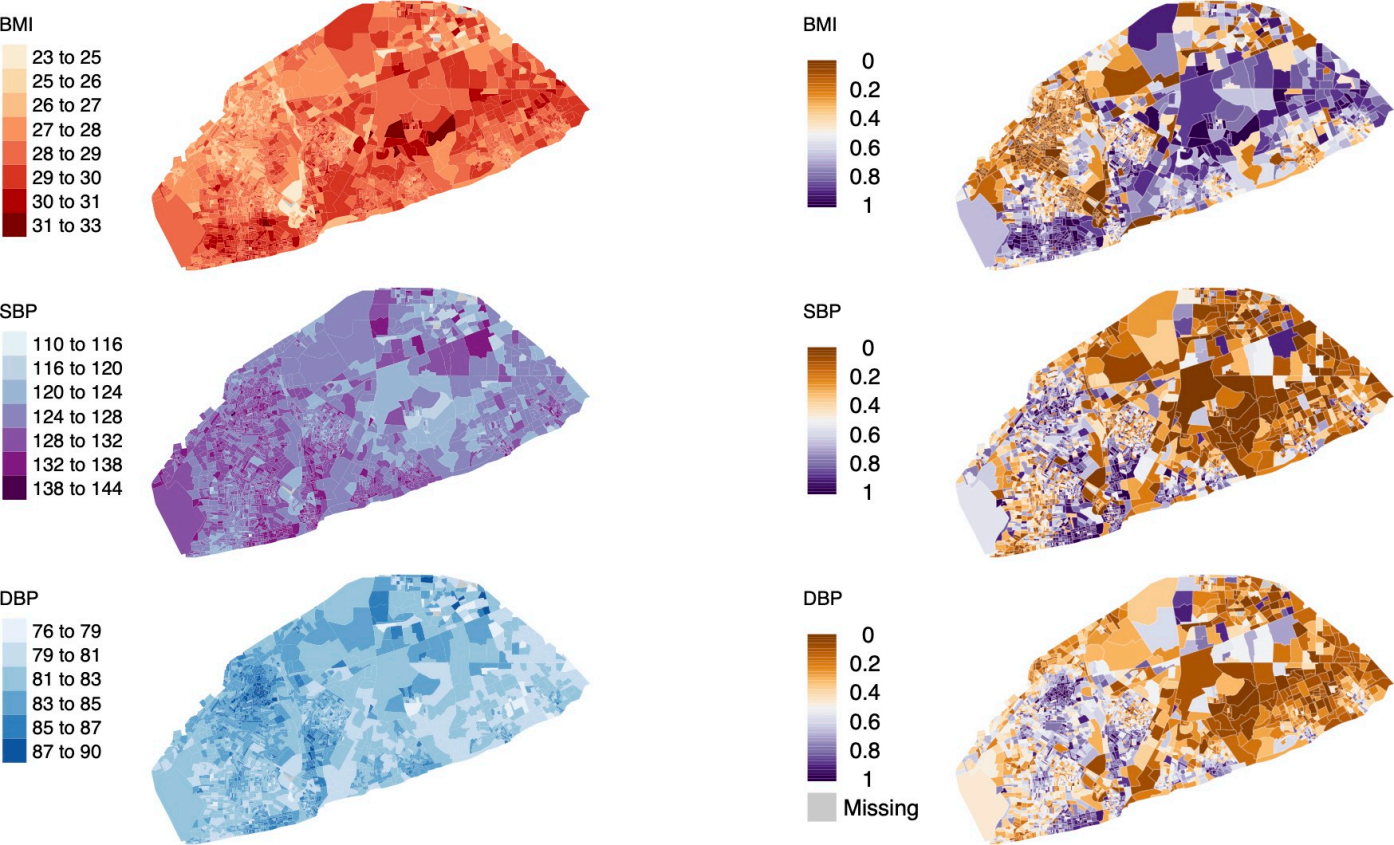
Spatial distribution of BP and BMI among women in Accra. The maps on the left represent the mean EA predicted BMI (kg/m^2^), SBP (mm Hg), and DBP (mm Hg) for adult women in Accra. The maps on the right represent the posterior probabilities that the mean EA BMI, SBP, and DBP values are greater than the mean values for all EAs. Predictions are missing for 4 EAs (grey) where the sample of women in the census were missing independent variable data. Accra and EA boundaries for the 2010 census are from the Ghana Statistical Service. BMI, body-mass index; BP, blood pressure; DBP, diastolic blood pressure; EA, enumeration area; SBP, systolic blood pressure.

**Fig 4:**
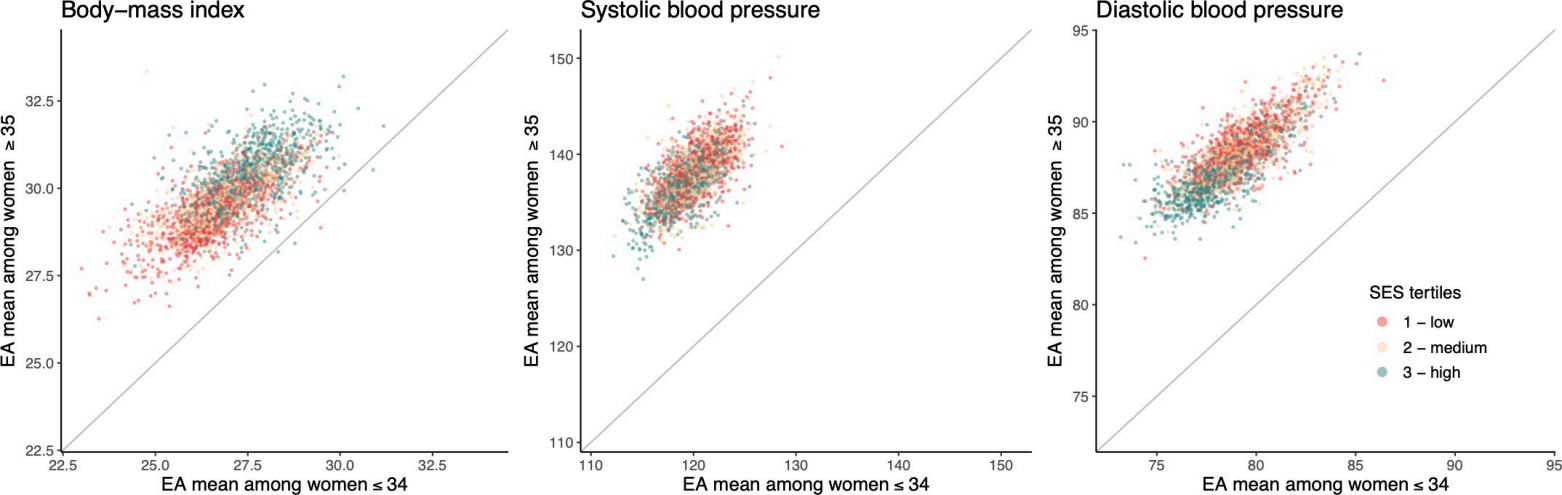
Relationship between mean EA BMI (kg/m^2^), SBP (mm Hg), and DBP (mm Hg) for women younger than 35 years and those 35 years and older in Accra in 2010. The position of each point along the x-axis and y-axis represents the mean BMI or BP among women in each age group within each EA. The colours of the points represent tertiles (33% increments) of mean EA relative SES based on household assets of women in the census. The SES measure, based on the first principal component (PC 1), was used as described in Methods. BMI, body-mass index; BP, blood pressure; DBP, diastolic blood pressure; EA, enumeration area; PC 1, principal component 1; SBP, systolic blood pressure; SES, socioeconomic status.

BMI and BP were spatially patterned across Accra. EAs with the highest mean BMI were mostly concentrated in the higher-income eastern areas of Accra (e.g., Cantonments, Osu, and Airport City) with some small clusters in the southwest (e.g., Chorkor and Dansoman) and near the University of Ghana (north central) (Fig 3). Conversely, the EAs with the highest predicted mean SBP were largely concentrated in the west of the city (e.g., Lapaz and Asylum Down) including the crowded old city core (Jamestown and Usshertown) and some large slum areas. There were also some concentrated pockets of EAs with higher mean SBP in the east, particularly in the southeast areas of Osu and Labadi. DBP had a similar spatial distribution as SBP, but the spatial patterns were more distinct (Figs C and D in S1 Text). The spatial distribution of BMI and BP in Accra was partly the result of the spatial distribution of some of the independent variables, such as ethnicity and relative SES as well as the spatial component in the model that captured spatial influences on the outcomes that are not due to the variables included in the model. While the median age of women in the 10% random sample of the census did vary across EAs, there was no spatial pattern (Fig E in S1 Text), and EA mean BMI had a similar spatial pattern among women younger and older than 35 years of age (Fig 4); this was the same for BP.

The MAE and the ME for the models with the core and extended variable set were similar (Table B in S1 Text). The MAE for the models with the core set of variables was 4.0 kg/m^2^ (BMI model), 12.5 mm Hg (SBP model), and 8.5 mm Hg (DBP model). The models’ MEs, a measure of bias, were close to zero. The lowest MAE was among the youngest age group (18- to 24-year-old women) and the highest among women older than 55 years (Table C in S1 Text).

## Discussion

We estimated that over a quarter of the adult women in Accra were classified as obese in 2010, and close to a quarter as having uncontrolled hypertension, with distinct spatial patterns between the two key NCD risk factors. Demographic (age, ethnicity, religion, and marriage status), socioeconomic (household assets and literacy), and behavioural factors (alcohol consumption and physical activity) had distinct associations with BMI and BP. We also observed that the variation of BMI and BP, and the prevalence of uncontrolled hypertension and obesity, across neighbourhoods within Accra was almost as large as variation across countries among women globally in 2010 [5–7].

Our estimate of the BMI of women in Accra in 2010 was similar to what was found in the 2006 WHO STEPs survey [37] and was higher than the estimated national average for Ghanaian women at that time [7], which is consistent with the evidence of higher BMI in urban compared with rural areas in Ghana [18] and SSA [11]. Similarly, the prevalence of women with uncontrolled hypertension in Accra was slightly higher than the crude prevalence among women in Ghana [5]. Accra is a city with large variations and inequalities in wealth, diversity of languages, ethnicities, and demographic makeup [19], largely due to migration into the city from other parts of Ghana and West Africa [19], which may partly underlie the spatial variation in BP and BMI.

Higher SES and literacy were associated with higher BMI and lower BP. This divergent trend between BP and BMI has been found in other studies in SSA [10,15–18,38–41]. Higher SES in SSA may lead to a higher caloric consumption and less energy expenditure in work and commute (e.g., commuting by private car [42]) that are risk factors for elevated BMI [15,43]. While perhaps a more diverse diet (e.g., higher consumption of fruits and vegetables) and better healthcare could facilitate lowering or control of BP at the same time [38,44].

Identifying as ethnically Ga was positively associated with increased BMI and BP, corroborating similar Accra- and Ghana-wide findings in the early 2000s [42,45]. This may be due to difference in diets and lifestyle compared with other ethnic groups. For example, within the Ga community in Accra, the primary income generating activities include fishing and food preparation and sales [46,47]. The Ga traditionally consume a local dish called Kenkey (fermented ground white corn), which is a high-energy food [39]. In addition, a previous study conducted in some traditional Ga communities in Accra (e.g., Jamestown and Usshertown) found that the local food environment was rich in high caloric foods and low in fresh fruits and vegetables [39]. Identifying as Muslim compared with other religious groups was also positively associated with BMI. In Accra, religion in some cases indicates ethnicity; for example, Muslim communities in Accra often originate from northern Ghana or other parts of West Africa and many speak a language called Hausa [20]. However, specific behavioural and nutritional differences between this group and other ethnicities have had limited study, although associations with lower physical activity levels have been found among young adults in Nigeria [48].

We found that BMI was positively associated with alcohol consumption among women in Accra, which has had mixed results in other studies in SSA [10,49]. However, we found that BP was negatively associated with alcohol consumption, which contrasts with a well-established association [50]. The negative association between BP and alcohol consumption among Accra woman could be due to the binary classification of general alcohol consumption ever, which may have categorised more women as exposed to this risk factor compared to measures based on frequency (e.g., number of drinks per day and type of drink) [51]. We also found that engaging in moderate and/or vigorous physical activity during a typical week was negatively associated with BP, which is consistent with other studies in SSA and elsewhere [52–55], which have found immediate and longer-term reductions in BP as a result of exercise. There is a well-established link between physical activity and BMI [10,39,49,55–58]. The association in our models was in the expected direction (i.e., negative association), but the 95% CI included the null association. Finally, women’s age was associated with both BP and BMI, consistent with vast literature in these areas [59].

Our study has a number of strengths. It presents a novel spatial analysis of BMI and BP levels among urban women in a region undergoing an epidemiological transition. We synthesised multiple data sources with a spatial model to estimate BMI and BP for all women in Accra and summarised at the small area level. Our study also has several limitations. We were restricted to analysis among women as the health survey was female focused. It is possible that the spatial patterns and factors associated with BMI and BP levels in Accra differ for men. While we were able to include many demographic, socioeconomic, and environmental variables in the prediction model, we were limited to what was included in both the WHSA surveys and the census and thus had to omit some of the behavioural variables. We did not have information on distance to local health centres or services, and how accessible they are, which could have an influence on some of the observed spatial patterns of BMI and BP via access to treatment and medication. Although we expect this impact on our study findings, if any, to be minimal as the size of the AMA is relatively small, and primary care, which is the level at which hypertension is often diagnosed and managed, is spread throughout the city [60]. As a result, household and individual socioeconomic factors, which were included in our model, are likely to have bigger influence on access to care and treatment, which remain much lower in SSA than in other regions [6,61,62]. Furthermore, we did not have robust information on typical or long-term dietary features, which are known determinants of BMI and BP [17,32]. We were also limited in our ability to accurately characterise noise and air pollution exposures, as we could only use proxy variables such as EA distance to major road and primary fuel used for cooking, which could explain the null associations (*P* < 0.95) with the outcomes. BP in particular is associated with air and noise pollution from road-traffic sources and air pollution from solid/biomass fuel use, in other studies [28,31]. We excluded pregnant women in the WHSA because pregnancy alters BMI and BP [63]. We did not have that same information for the census. Thus, the predictions should be interpreted as that of women in Accra at the time that they are not pregnant. Although in reality, we would expect approximately 6% of women in Accra to have altered BMI and BP due to pregnancy [64].

Finally, we were restricted to using the 2010 census and a health survey done around the same time as the census. It would be ideal to analyse the spatial patterns of NCD risk factors over time, using repeated population-based surveys and censuses. The 2020 census in Ghana has been delayed due to the Coronavirus Disease 2019 (COVID-19) pandemic, and its release date was unknown at the time of this article. Although the absolute levels of BMI and BP change over time, the spatial patterns are likely to do so more gradually because some dynamics are shared across population subgroups, while the others are distinct. We compared our 2010 estimates in AMA to the Demographic and Health Survey (DHS) among urban women in the Greater Accra Region conducted in 2014 (Table D in S1 Text) and found that the two sets of mean BMI were similar with BMI differences in different age groups ranging from −0.9 to 1.2 kg/m^2^, SBP differences from 7.8 to 12.1 mm Hg, and DBP differences from 3.0 to 3.8 mm Hg. The reasons for the observed differences may be because the DHS has a relatively small sample size (*n* = 416) and because the sample of women in the DHS was from the entire Greater Accra Region, and the census estimates were made for the AMA, which is more urban. Alternatively, differences could be due to actual changes in BMI and BP. For example, for BP, national level estimates have shown slight reductions from 2010 to 2015 by 1.7 mm Hg for age-standardised mean SBP and 0.5 mm Hg for age-standardised mean DBP [5]. This may have been due to changes in diet, e.g., more year-around availability of fruits and vegetables, or higher coverage of BP lowering treatment, which in Ghana increased more than most other countries in SSA [17]. Both BMI and BP have a well-known positive association with age [59]. As the age distribution of adult women in Accra is shifting towards older age over time—with median age among women 18 to 49 years old in the 2008 DHS as 29 (interquartile range (IQR): 23 to 36) and in the 2014 DHS as 32 (IQR: 25 to 38)—we would expect based on population age dynamics alone that BMI and BP would be higher on average in present day Accra than what we estimated. For example, national estimates indicate that age-standardised mean BMI of urban women in Ghana increased by 0.7 kg/m^2^ from 2010 to 2017 [11].

Cities in SSA are experiencing rapid changes due to migration into urban areas and transformations of the social, physical, and food environment, which can impact the development, progression, and distribution of NCDs and their risk factors [10,33,65,66]. Information on the spatial heterogeneity and inequalities in NCD risk factors, such as BMI and BP, can guide public health policy, actions, and interventions within SSA cities. Specifically, our results suggest that some of the actions to address elevated BMI and BP among women in Accra should be localised community-based approaches, such as encouraging health centres in specific neighbourhoods to prioritise measurement and provision of medicines, which are underutilised in SSA compared to other regions [6], or behavioural advice or working through community networks and groups in specific communities and social, ethnic, and religious groups to encourage care seeking and behavioural change.

## Supporting information

S1 STROBE Checklist STROBE Checklist. STROBE, Strengthening the Reporting of Observational Studies in Epidemiology.
(DOC)

S1 Text **Supporting information Figs A–F and Tables A–D. Fig A:** Factor loadings for PC 1 and PC 2 for the 2010 census. **Fig B:** Associations of BP and BMI with demographic, socioeconomic, and environmental factors. **Fig C:** Geographic areas in Accra. **Fig D:** Cumulative densities of predicted BMI (kg/m^2^), SBP (mm Hg), and DBP (mm Hg) for census women stratified by older (35 and older) and younger (less than 35 years old) age groups. **Fig E:** Median age for adult (≥18 years) women in the 10% random sample of the census included in the analysis within each EA. **Fig F:** Spatial distribution of the prevalence of diabetes in Accra from the WHSA 2008 to 2009. **Table A:** Summaries of SBP, DBP, and BMI among adult nonpregnant women in the WHSA (2008 to 2009). **Table B:** Comparison of prediction error between the model predictions with the extended and core variable set. **Table C:** Model prediction error using the core set of variables, by age group. **Table D:** Mean BMI and BP for women in the DHS 2014 survey in urban areas in Greater Accra. BMI, body-mass index; BP, blood pressure; DBP, diastolic blood pressure; DHS, Demographic and Health Survey; EA, enumeration area; PC, principal component; SBP, systolic blood pressure.
(DOCX)

## Abbreviations:

95% CrI: 95% credible interval
AMA: Accra Metropolitan Area
BMI: body-mass index
BP: blood pressure
CAR: conditional autoregressive
COVID-19: Coronavirus Disease 2019
CVD: cardiovascular disease
DBP: diastolic blood pressure
DHS: Demographic and Health Survey
EA: enumeration area
IQR: interquartile range
MAE: median absolute error
MCMC: Markov chain Monte Carlo
ME: mean error
NCD: noncommunicable disease
PP: posterior probability
SBP: systolic blood pressure
SES: socioeconomic status
SSA: sub-Saharan Africa
STROBE: Strengthening the Reporting of Observational Studies in Epidemiology
WHSA: Women’s Health Study of Accra
WHO: World Health Organization
