# Supplementary material for: Small area variations and factors associated with blood pressure and body-mass index in adult women in Accra, Ghana: Bayesian spatial analysis of a representative population survey and census data
Source: PLoS Med. 2021 Nov 11;18(11):e1003850. doi: 10.1371/journal.pmed.1003850 (PMC8584976; doi:10.1371/journal.pmed.1003850)
Supplement: S1 STROBE Checklist — STROBE, Strengthening the Reporting of Observational Studies in Epidemiology. (DOC) [file pmed.1003850.s001.doc]

STROBE Statement—Checklist of items that should be included in reports of ***cross-sectional studies***

Page numbers refer to the marked-up manuscript

|  | Item No | Recommendation |
| --- | --- | --- |
| **Title and abstract** | 1 | (*a*) Indicate the study’s design with a commonly used term in the title or the abstract  **Title and Abstract (Methods and Findings)** |
|  |  | (*b*) Provide in the abstract an informative and balanced summary of what was done and what was found **Abstract: *Methods and Findings* and Abstract Summary** |
| Introduction | | |
| Background/rationale | 2 | Explain the scientific background and rationale for the investigation being reported  **Introduction (Paragraphs 1, 2, and 3)** |
| Objectives | 3 | State specific objectives, including any prespecified hypotheses **Introduction Paragraph 3 (Not applicable for hypothesis)** |
| Methods | | |
| Study design | 4 | Present key elements of study design early in the paper. **Introduction (Paragraph 3), Methods: *Data sources* (Paragraph 1) and Methods: *Statistical analysis* (Paragraph 1)** |
| Setting | 5 | Describe the setting, locations, and relevant dates, including periods of recruitment, exposure, follow-up, and data collection  **Methods: *Study area* and population and Methods: *Data sources*** |
| Participants | 6 | (*a*) Give the eligibility criteria, and the sources and methods of selection of participants **Methods: *Data sources* (Paragraph 2 and 4)** |
| Variables | 7 | Clearly define all outcomes, exposures, predictors, potential confounders, and effect modifiers. Give diagnostic criteria, if applicable  **Methods: *Data sources* (Paragraph 3), Methods: *Statistical analysis* (Paragraph 2, 3, 6, 7),** |
| Data sources/ measurement | 8* | For each variable of interest, give sources of data and details of methods of assessment (measurement). Describe comparability of assessment methods if there is more than one group  **Sources - Methods: *Data sources* (Paragraph 1, 2, 4, 5)**  **Measurement – Methods: *Data sources* (Paragraph 3), Methods: *Statistical analysis* (Paragraph 6), Appendix Figure 1** |
| Bias | 9 | Describe any efforts to address potential sources of bias  **Methods: *Data sources* (Paragraph 3) -** **we removed women who were pregnant at the time of survey as their BP and BMI would be affected by it.**  **Methods: *Statistical analysis* (Paragraph 7) - we used objective anthropometric measurements (BMI, BP) to estimate the prevalence of obesity and uncontrolled hypertension to address potential recall or self-report bias.** |
| Study size | 10 | Explain how the study size was arrived at  **Methods: *Data sources* (Paragraph 2) - based on a power calculation described in Hill et al. 2007.** |
| Quantitative variables | 11 | Explain how quantitative variables were handled in the analyses. If applicable, describe which groupings were chosen and why  **Methods: *Statistical analysis* (Paragraph 2, 6, 7)** |
| Statistical methods | 12 | (*a*) Describe all statistical methods, including those used to control for confounding  **Methods: *Statistical analysis* (all paragraphs)** |
|  |  | (*b*) Describe any methods used to examine subgroups and interactions  **Methods: *Statistical analysis* (Paragraph 7) -** **subgroup by age** |
|  |  | (*c*) Explain how missing data were addressed  **Methods: *Statistical analysis* (Paragraph 2)** |
|  |  | (*d*) If applicable, describe analytical methods taking account of sampling strategy **NA** |
|  |  | (*e*) Describe any sensitivity analyses **NA** |
| Results | | |
| Participants | 13* | (a) Report numbers of individuals at each stage of study—eg numbers potentially eligible, examined for eligibility, confirmed eligible, included in the study, completing follow-up, and analysed  **Methods: *Data sources* (Paragraph 2, 4) - enrolled in study and response rate**  **Methods: *Statistical analysis* (Paragraph 2) - missing data**  **Table 1 – included in analysis** |
|  |  | (b) Give reasons for non-participation at each stage **NA (Using WHSA survey with 99% response and census)** |
|  |  | (c) Consider use of a flow diagram **NA (using secondary data where this information has been reported extensively in previous publications, cited in Methods: *Data sources* (Paragraph 2))** |
| Descriptive data | 14* | (a) Give characteristics of study participants (eg demographic, clinical, social) and information on exposures and potential confounders  **Table 1** |
|  |  | (b) Indicate number of participants with missing data for each variable of interest **Methods: *Statistical analysis* (Paragraph 2)** |
| Outcome data | 15* | Report numbers of outcome events or summary measures  **Table 1** |
| Main results | 16 | (*a*) Give unadjusted estimates and, if applicable, confounder-adjusted estimates and their precision (eg, 95% confidence interval). Make clear which confounders were adjusted for and why they were included  **NA (Bayesian credible intervals are presented wherever relevant)** |
|  |  | (*b*) Report category boundaries when continuous variables were categorized  **Table 1** |
|  |  | (*c*) If relevant, consider translating estimates of relative risk into absolute risk for a meaningful time period **NA** |
| Other analyses | 17 | Report other analyses done—eg analyses of subgroups and interactions, and sensitivity analyses **NA** |
| Discussion | | |
| Key results | 18 | Summarise key results with reference to study objectives  **Discussion (Paragraph 1)** |
| Limitations | 19 | Discuss limitations of the study, taking into account sources of potential bias or imprecision. Discuss both direction and magnitude of any potential bias  **Discussion (Paragraph 6-7)** |
| Interpretation | 20 | Give a cautious overall interpretation of results considering objectives, limitations, multiplicity of analyses, results from similar studies, and other relevant evidence  **Discussion (Paragraph 1-5, 8)** |
| Generalisability | 21 | Discuss the generalisability (external validity) of the study results  **Discussion (Paragraph 8)** |
| Other information | | |
| Funding | 22 | Give the source of funding and the role of the funders for the present study and, if applicable, for the original study on which the present article is based  **Acknowledgements** |

*Give information separately for exposed and unexposed groups.

**Note:** An Explanation and Elaboration article discusses each checklist item and gives methodological background and published examples of transparent reporting. The STROBE checklist is best used in conjunction with this article (freely available on the Web sites of PLoS Medicine at http://www.plosmedicine.org/, Annals of Internal Medicine at http://www.annals.org/, and Epidemiology at http://www.epidem.com/). Information on the STROBE Initiative is available at www.strobe-statement.org.
