# Supplementary material for: Small area variations and factors associated with blood pressure and body-mass index in adult women in Accra, Ghana: Bayesian spatial analysis of a representative population survey and census data
Source: PLoS Med. 2021 Nov 11;18(11):e1003850. doi: 10.1371/journal.pmed.1003850 (PMC8584976; doi:10.1371/journal.pmed.1003850)
Supplement: S1 Text — Supporting information Figs A–F and Tables A–D. Fig A: Factor loadings for PC 1 and PC 2 for the 2010 census. Fig B: Associations of BP and BMI with demographic, socioeconomic, and environmental factors. Fig C: Geographic areas in Accra. Fig D: Cumulative densities of predicted BMI (kg/m2), SBP (mm Hg), and DBP (mm Hg) for census women stratified by older (35 and older) and younger (less than 35 years old) age groups. Fig E: Median age for adult (≥18 years) women in the 10% random sample of the census included in the analysis within each EA. Fig F: Spatial distribution of the prevalence of diabetes in Accra from the WHSA 2008 to 2009. Table A: Summaries of SBP, DBP, and BMI among adult nonpregnant women in the WHSA (2008 to 2009). Table B: Comparison of prediction error between the model predictions with the extended and core variable set. Table C: Model prediction error using the core set of variables, by age group. Table D: Mean BMI and BP for women in the DHS 2014 survey in urban areas in Greater Accra. BMI, body-mass index; BP, blood pressure; DBP, diastolic blood pressure; DHS, Demographic and Health Survey; EA, enumeration area; PC, principal component; SBP, systolic blood pressure. (DOCX) [file pmed.1003850.s002.docx]

**Supplementary Information**

**Small-area variations and factors associated with blood pressure and body-mass index in adult women in Accra, Ghana: Bayesian spatial analysis of a representative population survey and census data**

Contents

[Fig A. Factor loadings for principal component 1 (PC 1) and 2 (PC 2) for the 2010 census. 2](#_Toc85204947)

[Fig B. Associations of blood pressure and body-mass index with demographic, socioeconomic and environmental factors. 4](#_Toc85204948)

[Fig C. Geographic areas in Accra. 5](#_Toc85204949)

[Fig D. Cumulative densities of predicted body-mass index (BMI, kg/m2), systolic blood pressure (SBP, mmHg), and diastolic blood (DBP, mmHg) pressure for census women stratified by older (35 and older) and younger (less than 35 years old) age groups. 6](#_Toc85204950)

[Fig E. Median age for adult (≥18 years) women in the 10% random sample of the census included in the analysis within each enumeration area (EA). 7](#_Toc85204951)

[Fig F. Spatial distribution of the prevalence of diabetes in Accra from the Women’s Health Study of Accra 2008-2009. 8](#_Toc85204952)

[Table A. Summaries of systolic blood pressure, diastolic blood pressure, and body-mass index among adult non-pregnant women in the WHSA (2008-2009). 9](#_Toc85204953)

[Table B. Comparison of prediction error between the model predictions with the extended and core variable set. 10](#_Toc85204954)

[Table C. Model prediction error using the core set of variables, by age group. 10](#_Toc85204955)

[Table D. Mean body-mass index and blood pressure for women in the Demographic and Health (DHS)1 2014 survey in urban areas in Greater Accra 11](#_Toc85204956)


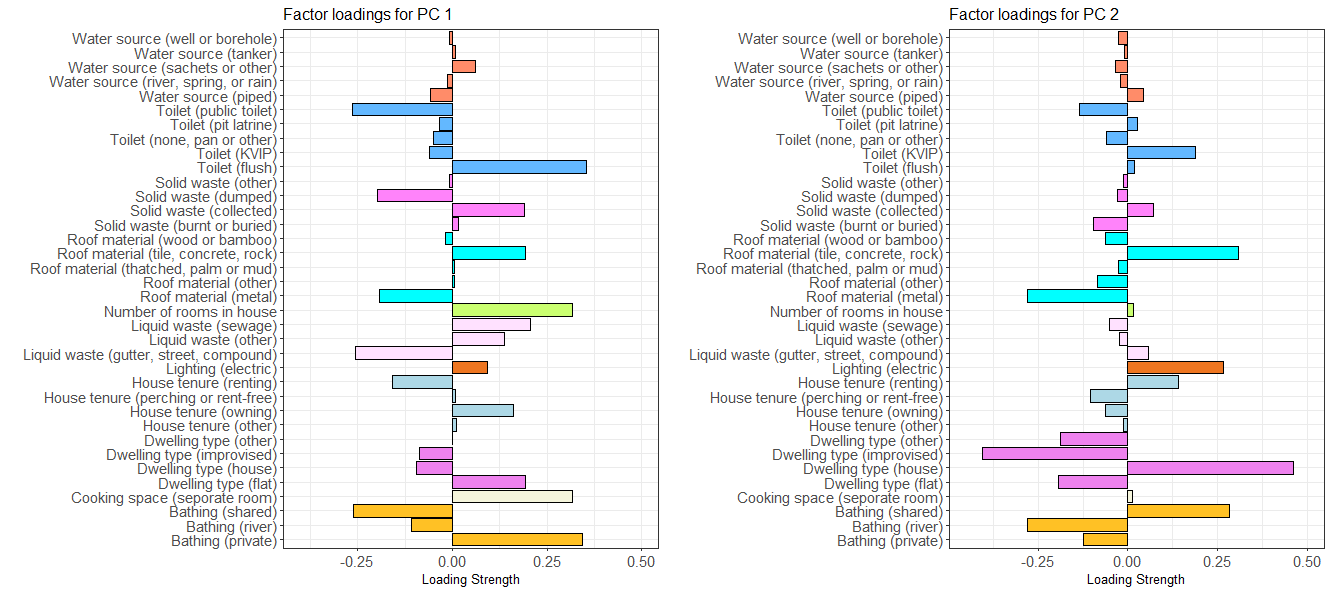


**Fig A. Factor loadings for principal component 1 (PC 1) and 2 (PC 2) for the 2010 census.** Coloured bars indicate variables within the same category.

Variables representing socio-economic status (SES) were constructed for each woman in the Women’s Health Survey of Accra (WHSA) and census using principal component analysis (PCA) of household asset-based indicators which included fuel used for lighting, dwelling type and tenure, cooking space, roof materials, bathing facilities, solid and liquid waste disposal, water source, toilet facilities, and the number of rooms in the household. The PCA was run on the 2010 census and the factor loadings were applied to the WHSA. We retained the 1^st^ and 2^nd^ principal components (PC 1 and PC 2) because they represent the linear combinations of the variables that explained the highest and second highest share of variance. The SES measure (SES PC 1) constructed from the factor loadings from the 1^st^ principal component contained the clearest representation of household assets and wealth. The factor loadings with the most strength in the positive direction for PC 1 included dwellings that are owned and have a larger number of rooms, a separate space for cooking, household dwellings that have permanent roof structures (tiles, concrete, rock), flush toilet infrastructure, private bathing facilities, and solid and liquid waste that is collected. Negative PC 1 values are impacted the most by household dwellings that are rented, with metal roofs, have shared/public bathing and toilet facilities, and when liquid and solid waste is disposed of in the gutter or street. Factor loadings from PC 2 represented a slightly more complex representation of household assets. Factor loadings in the positive direction represented households with rented dwellings that had some permanent structures (e.g., roofs with tiles/concrete/rock) and modern fuel for lighting the house (electricity), but some communal facilities such as shared bathing facilities and Kumasi Ventilated Improved Pit (KVIP) toilets.

**
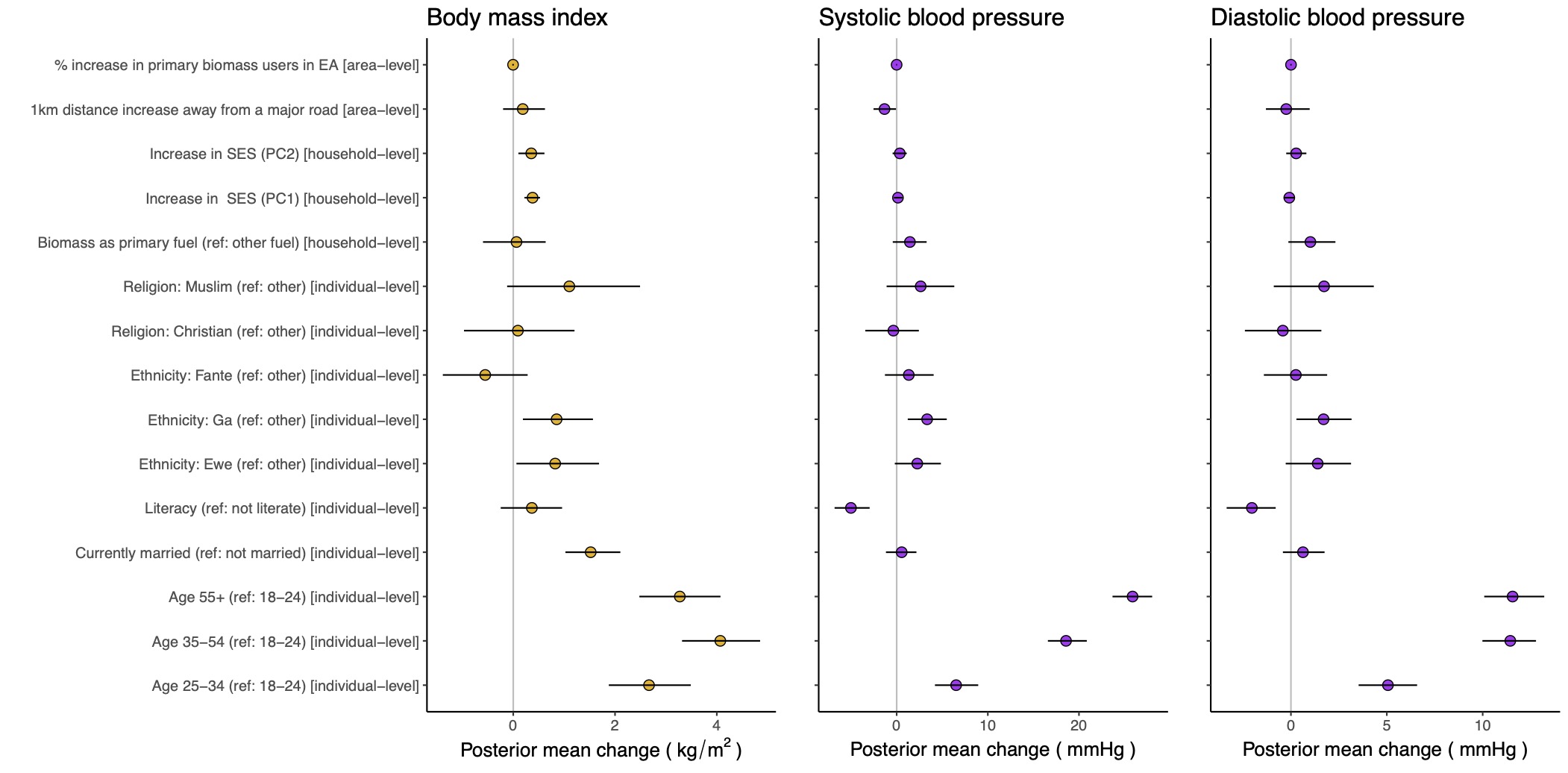
**

**Fig B. Associations of blood pressure and body-mass index with demographic, socioeconomic and environmental factors.** The variable set includes the core variables used for prediction with the census. For each variable, the figure shows the posterior mean association and the 95% credible interval around the mean. *Legend:* *Household SES PC1 and 2: household asset-based wealth index principal components 1 and 2; EA: enumeration area.*


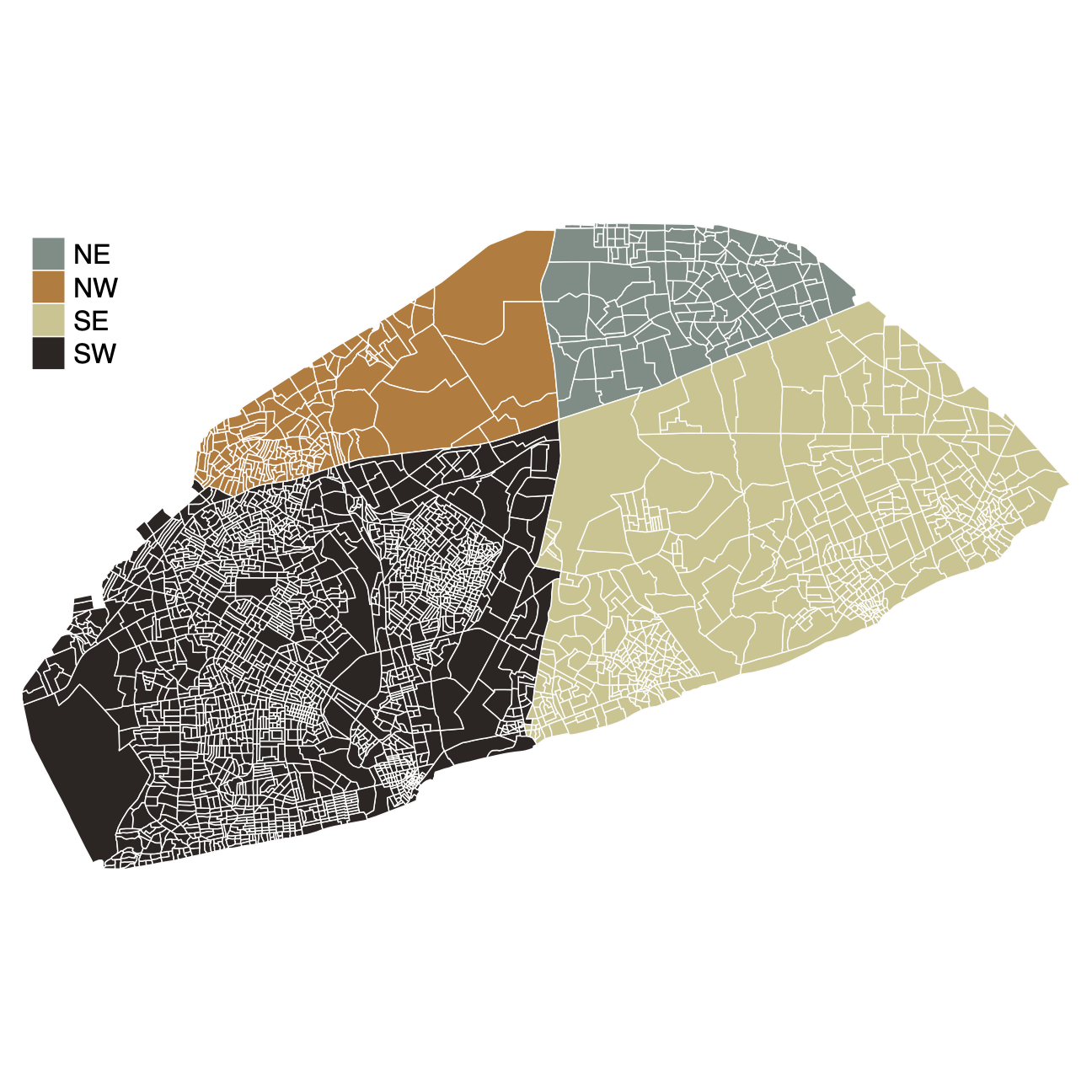


**Fig C. Geographic areas in Accra.** North-South and East-West areas are separated by major highways running east-west and north-south and enumeration area boundaries. NE: North East; NW: North West; SE: South East; SW: South West. Accra and EA boundaries are from the Ghana Statistical Service.

**
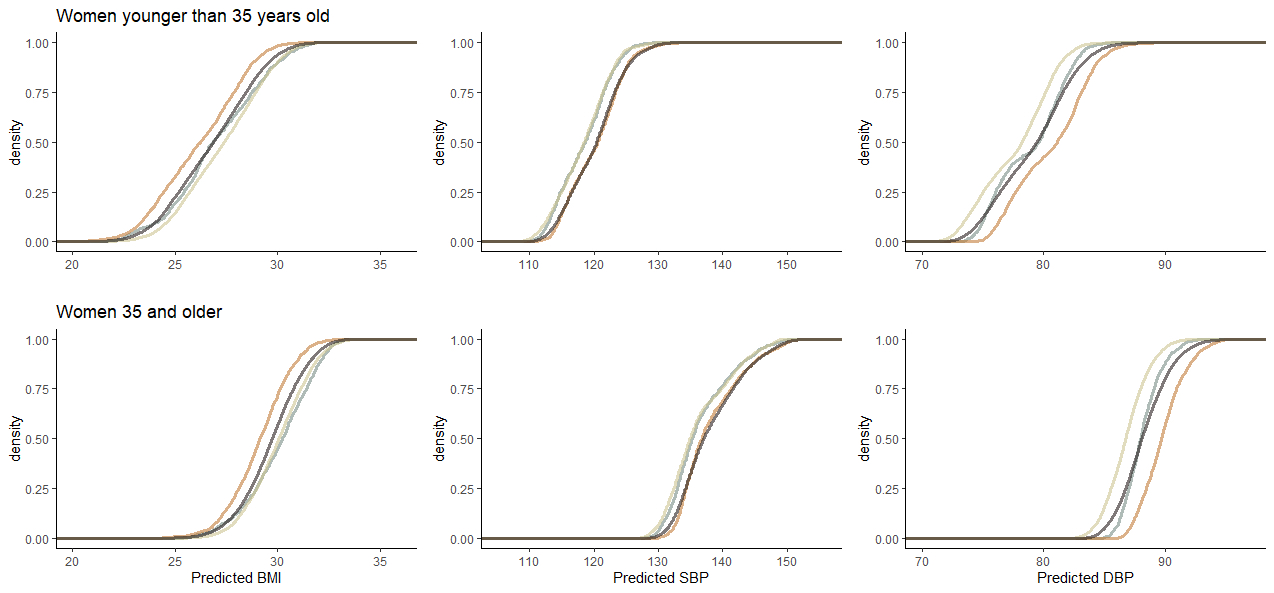
**

**Fig D. Cumulative densities of predicted body-mass index (BMI, kg/m2), systolic blood pressure (SBP, mmHg), and diastolic blood (DBP, mmHg) pressure for census women stratified by older (35 and older) and younger (less than 35 years old) age groups.** NE: North East; NW: North West; SE: South East; SW: South West.

**
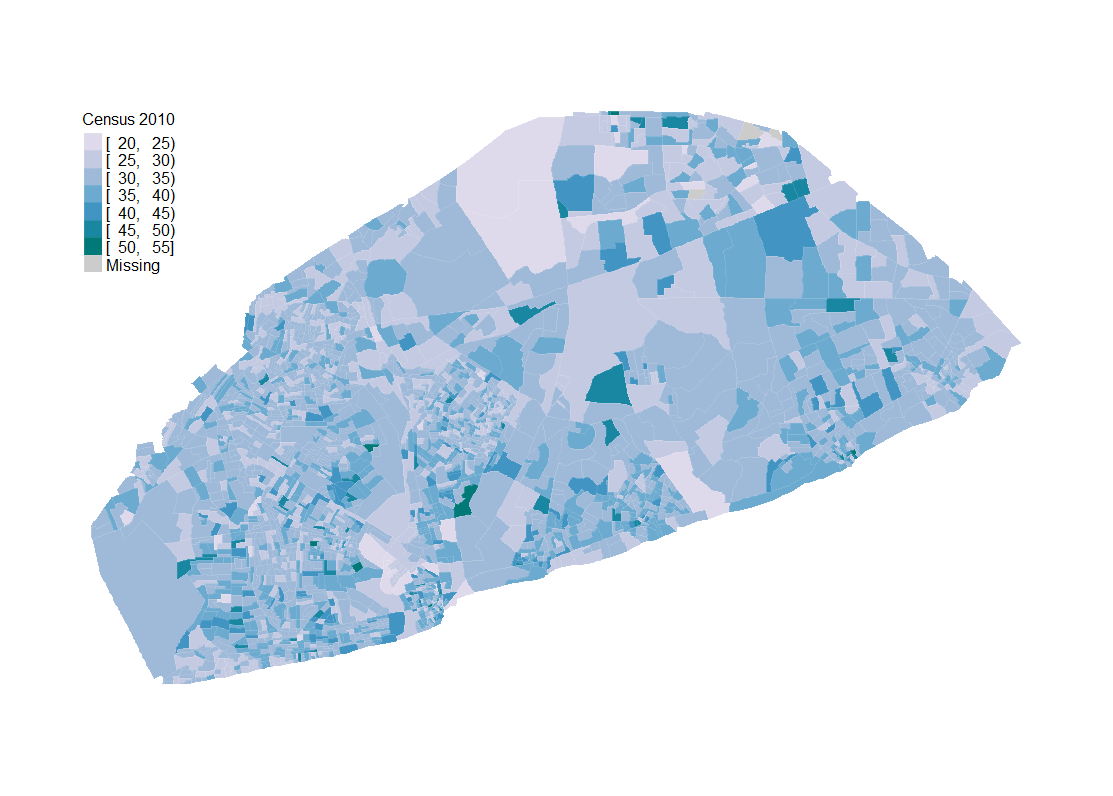
**

**Fig E. Median age for adult (≥18 years) women in the 10% random sample of the census included in the analysis within each enumeration area (EA).** The large EA in the top centre of Accra represents the University of Ghana campus which explains the low median age of adult women in the EA. Accra and EA boundaries are from the Ghana Statistical Service.

**
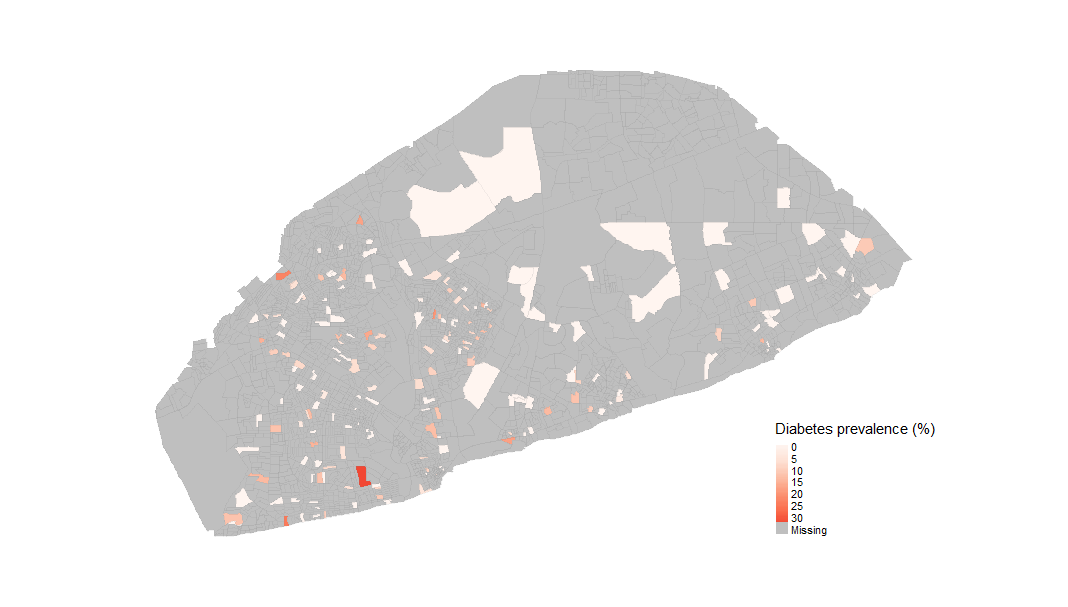
**

**Fig F. Spatial distribution of the prevalence of diabetes in Accra from the Women’s Health Study of Accra 2008-2009.** Accra and EA boundaries are from the Ghana Statistical Service.

125 (5%) women self-reported as being diagnosed with diabetes in 12 months from the WHSA survey (2008-2009). The median age of women with diabetes was 65 (range: 32 – 99 years). Figure 6 shows the spatial distribution of the prevalence of diabetes in Accra. This is likely an underestimate of the total prevalence because many people with diabetes have not had a diagnosis. This contrasts with the estimates of uncontrolled hypertension which are based on actual blood pressure measurement.

**Table A. Summaries of systolic blood pressure, diastolic blood pressure, and body-mass index among adult non-pregnant women in the WHSA (2008-2009).** For each variable the table shows the mean and standard deviation.

| Age range | Systolic blood pressure  (mmHg) | Diastolic blood pressure (mmHg) | Body-mass index  (kg/m^2^) |
| --- | --- | --- | --- |
| All ages | 130.7 (23.9) | 83.9 (14.7) | 28.4 (6.9) |
| 18-24 | 117.0 (13.9) | 76.5 (11.6) | 25.8 (5.8) |
| 25-34 | 123.7 (18.5) | 81.9 (13.2) | 28.8 (6.7) |
| 35-54 | 136.7 (23.9) | 88.6 (14.9) | 30.3 (7.0) |
| 55+ | 145.5 (26.1) | 89.1 (15.2) | 29.1 (7.2) |

**Table B. Comparison of prediction error between the model predictions with the extended and core variable set.** The median absolute error (MAE) and mean error (ME) of predictions. WHSA: Women’s Health Survey of Accra.

|  | Core variable set | | | Extended variable set | | |
| --- | --- | --- | --- | --- | --- | --- |
|  |  | Median absolute error (MAE) | Mean error (ME) |  | Median absolute error (MAE) | Mean error (ME) |
| WHSA (2008/2009) |  |  |  |  |  |  |
| Body-mass index (kg/m^2^) |  | 4.02 | 0.003 |  | 3.98 | -0.021 |
| Systolic blood pressure (mmHg) |  | 12.48 | 0.011 |  | 11.80 | -0.005 |
| Diastolic blood pressure (mmHg) |  | 8.54 | -0.001 |  | 8.51 | -0.0006 |

# **Table C. Model prediction error using the core set of variables, by age group.**

|  | Median absolute error (Interquartile range) | | | |
| --- | --- | --- | --- | --- |
|  | **Age 18-24** | **Age 25-34** | **Age 35-54** | **Age 55+** |
| Body-mass index (kg/m^2^) | 3.4 (1.7, 5.5) | 4.2 (2.1, 6.8) | 4.1 (2.1, 6.9) | 4.6 (2.4, 7.6) |
| Systolic blood pressure (mmHg) | 8.6 (4.1, 14.6) | 10.7 (4.5, 18.2) | 16.1 (7.5, 25.5) | 17.1 (8.0, 27.8) |
| Diastolic blood pressure (mmHg) | 7.5 (3.4, 12.7) | 8.2 (3.4, 13.8) | 9.7 (4.0, 16.0) | 9.7 (4.4, 16.1) |

**Table D. Mean body-mass index and blood pressure for women in the Demographic and Health (DHS)1 2014 survey in urban areas in Greater Accra***. Only women 15-49 included in survey.

| Age | Number of women (%) | Body-mass index  kg/m^2^ | Systolic blood pressure  mmHg | Diastolic blood pressure  mmHg |
| --- | --- | --- | --- | --- |
| 18-24 | 96 (23) | 24.0 | 108.1 | 72.8 |
| 25-34 | 165 (40) | 27.0 | 113.9 | 78.2 |
| 35-49 | 155 (37) | 30.9 | 123.0 | 83.9 |

* Greater Accra Region (GAR) restricted to areas classified as urban in dataset (89% of DHS adult women in GAR).

**Reference**

(1) Ghana Statistical Service. *Ghana Demographic and Health Survey*; 2014.
